# Supplementary material for: Protein and Amino Acid Supplementation Among Recreational Gym Goers and Associated Factors—An Exploratory Study
Source: J Funct Morphol Kinesiol. 2025 Jun 28;10(3):248. doi: 10.3390/jfmk10030248 (PMC12286015; doi:10.3390/jfmk10030248)

Questionnaire

We note that the data obtained from the application of this questionnaire will be statistically processed and will not be used in its original form. For the statistical use of the data, informed consent was obtained from each subject. Any information that could lead to the identification of the subjects will not be made public, maintaining the confidentiality of personal data.

1. Sex (Male and Female) and Age (18-30, 31-40, 41-50, 51-60);
2. Body weight type (based on BMI calculation): UW, NW, OW, obese
3. Educational level (high school, graduate, master/doctorate)
4. Monthly income (<2000, 2000-4000, 4000-6000, >6000 RON)
5. Daily working time (1-4 h, 4-8 h, >8 h)
6. Working activity (office, physical activity, office+physical activity)
7. Smoking status (Yes/No)
8. Alcohol consumption frequency (never, daily, weekly, monthly, occasionally)
9. Daily meal frequency (3 meals/day, 2 meals/day, 3 meals + 2 snacks, intermittent fasting)
10. Diet type (balanced, hyperprotein, vegetarian, low-carb)
11. Daily calorie intake (I don't know, <1000, 1001-1500, 1501-2000, 2001-2500, 2501-3000, 3001-3500, >3500)
12. Daily protein intake (I don't know, <50, 51-100, 101-150, 151-200, 201-250, >250)
13. Gym-goer status (<1 month, 1-6 months, 6-12 months, > 1 year)
14. Gym weekly frequency (< 3 times/weekly, 3-4 times/weekly, ≥ 5 times/weekly)
15. Gym training duration (<1 h, 1-2 h, >2 h)
16. Training type (Cardio, Force, Cardio+Force)
17. Training scope (muscle mass tonus, muscle mass growing, weight loss, competition)
18. Protein and amino acid supplements consumers (protein, creatine, L-carnitine, triple combination)
19. NS use period (constantly, muscle mass growing period, muscle definition period)
20. NS consumption period (< 1 year, 1-3 years, >3 years)
21. NS frequency (daily, training days only)
22. The main reason for NS consumption (weight loss, fat burning, physical effort capacity, physical effort recovery, muscle mass growing)
23. Potential side effects claimed (diarrhea, kidney damage, liver damage, muscle cramps, nausea, weight gain, no side effects)
24. Protein daily dose (20 g, 40 g, 60 g)
25. Creatine daily dose (1-5 g, 6-10 g)
26. L-carnitine daily dose (1 g, 2 g, 3 g, 5-10 g)

Reliability Analysis:

Cronbach's alpha statistics :

Cronbach's alpha	Standardized Cronbach's Alpha
0.947	0.946

Guttman statistics:

Guttman L1	Guttman L2	Guttman L3	Guttman L4	Guttman L5	Guttman L6
0.910	0.963	0.947	0.999	0.941	1.000

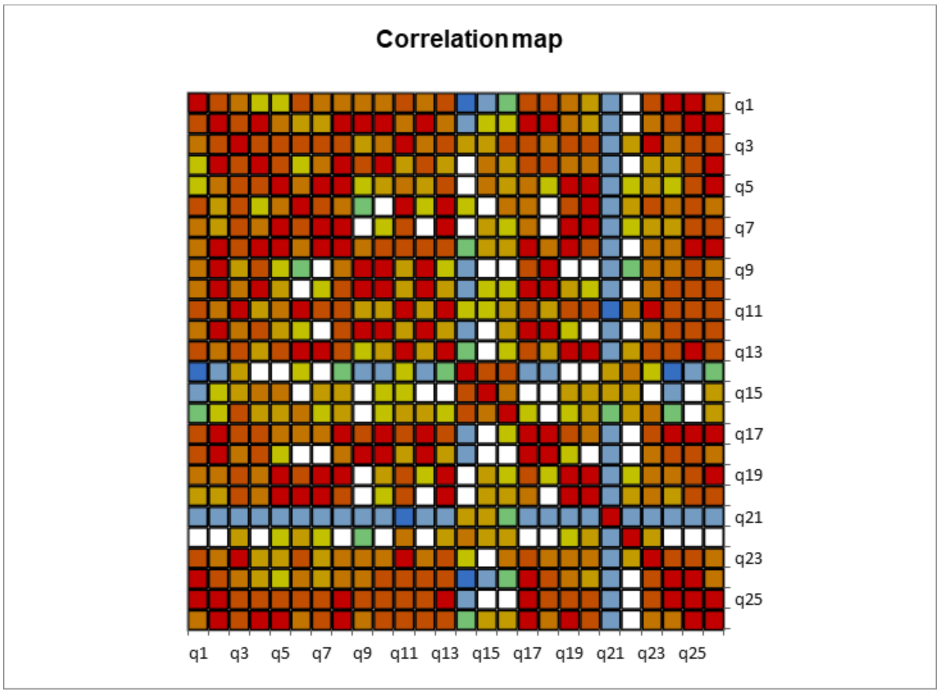

Supplement: Supplementary file 1 [file jfmk-10-00248-s001.zip › jfmk-3678035-supplementary.pdf]
